# Supplementary material for: The interactome of intact mitochondria by cross-linking mass spectrometry provides evidence for coexisting respiratory supercomplexes
Source: Mol Cell Proteomics. 2017 Dec 8;17(2):216–32. doi: 10.1074/mcp.RA117.000470 (PMC5795388; doi:10.1074/mcp.RA117.000470)
Supplement: Supplemental Data [file supp_RA117.000470_133922_0_supp_23978_fzffwf.zip › spectra_annotation/mito_DR_spectra_annotation/367-1-13-1-4-1.pdf]

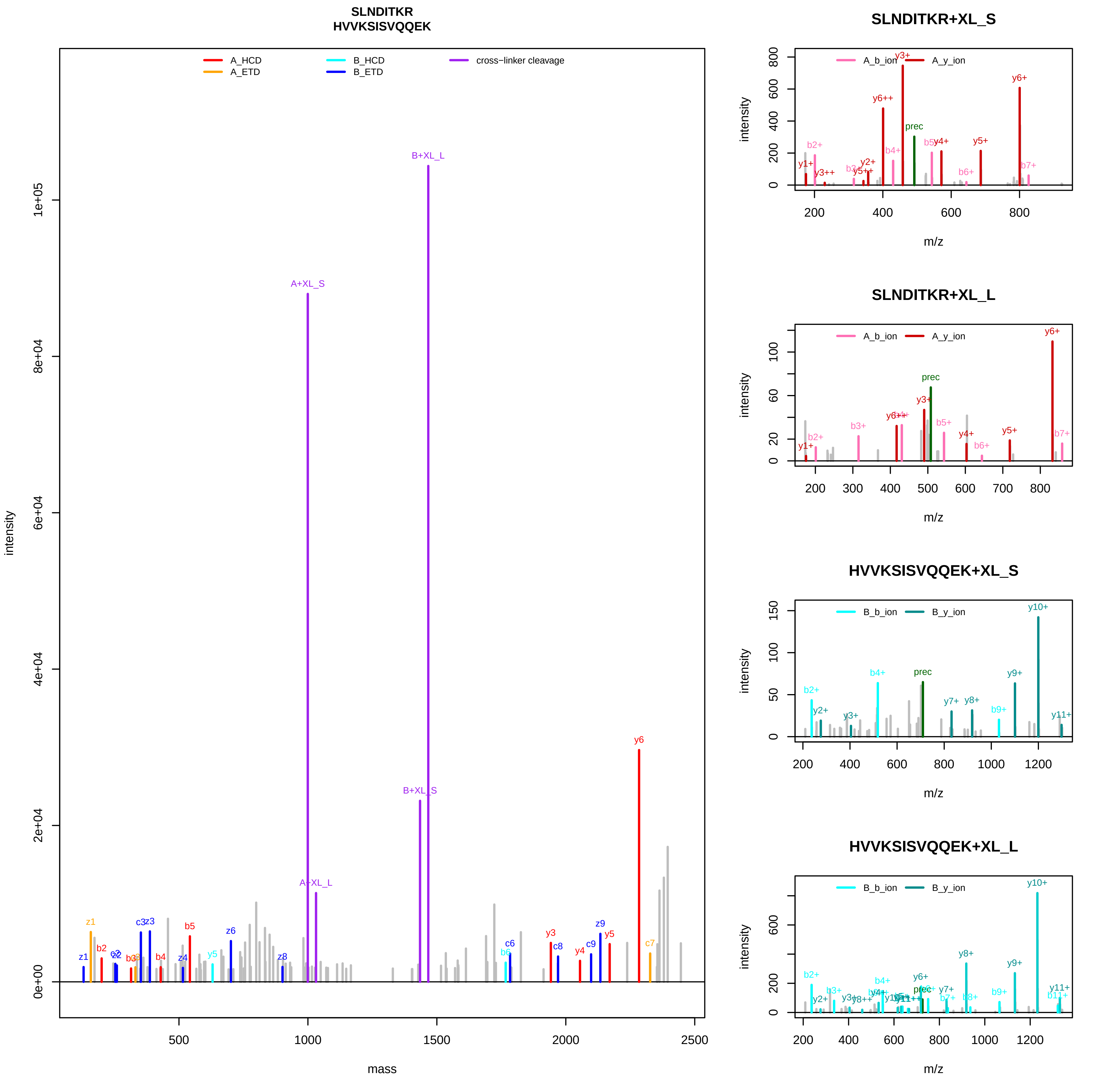
SLNDITKR
HVVKSISVQQEK
A_HCD
A_ETD
B_HCD
B_ETD
cross−linker cleavage
intensity
mass
0e+00
2e+04
4e+04
6e+04
8e+04
1e+05
500
1000
1500
2000
2500
B+XL_L
A+XL_S
B+XL_S
A+XL_L
y6
y3
y4
y5
c8
c9
z9
c7
c6
b6
z8
z6
y5
z4
b5
b4
c3
z3
b3
b2
c2
z2
z1
SLNDITKR+XL_S
SLNDITKR+XL_L
HVVKSISVQQEK+XL_S
HVVKSISVQQEK+XL_L
A_b_ion
A_y_ion
B_b_ion
B_y_ion
prec
y10+
y9+
y8+
y7+
y6+
y4+
y3+
y2+
y1+
b2+
b4+
b5+
b6+
b7+
b9+
y11+
m/z
